# Trends in prevalence of arthritis by race among adults in the United States, 2011–2018

**DOI:** 10.1186/s12889-024-18966-0

**Published:** 2024-06-05

**Authors:** Shenghao Xu, Xianyue Shen, Bo Chen, Yingqiao Sun, Xiongfeng Tang, Jianlin Xiao, Yanguo Qin

**Affiliations:** 1 Department of Orthopedics, The Second Hospital of Jilin University, Ziqiang St No. 218, Changchun, Jilin Province 130041 China; 2 Joint International Research Laboratory of Ageing Active Strategy and Bionic Health in Northeast Asia of Ministry of Education, Jilin University, Changchun, Jilin Province 130041 China; 3 Department of Orthopedics, China-Japan Union Hospital of Jilin University, Xiantai St No.126, Changchun, Jilin Province 130033 China; 4 Department of Orthopedics, The First Affiliated Hospital of University of Science and Technology of China, Lujiang Road No. 17, Hefei, Anhui Province 230001 China

**Keywords:** Arthritis, Trend, Prevalence, NHANES

## Abstract

**Background:**

There is currently a lack of comprehensive prevalence information on arthritis and its various classifications among adults in the U.S., particularly given the notable absence of detailed data regarding the Asian population. We examined the trends in the prevalence of arthritis, including osteoarthritis (OA), rheumatoid arthritis (RA), psoriatic arthritis (PsA), and other types of arthritis, among U.S. adults by race between 2011 and 2018.

**Methods:**

We analyzed data from the National Health and Nutrition Examination Survey (NHANES), spanning from 2011 to 2018. Our study focused on a nationally representative sample of U.S. adults aged 20 and older. Participants who answered “y es” to the research question “Doctors ever said you had arthritis?” were classified as having arthritis. Further classification into specific diseases was based on responses to the question “Which type of arthritis was it?” with options including “OA or degenerative arthritis, ” “RA, ” “PsA, ” or “Other. ”

**Results:**

We analyzed 22,566 participants from NHANES (2011–2018), averaging 44.8 years, including 10,927 males. The overall arthritis prevalence rose significantly from 22.98% (95% CI: 21.47–24.55%) in 2011–12 to 27.95% (95% CI: 26.20–29.76%) in 2017–18 (*P* for trend < 0.001). OA increased from 12.02% (95% CI: 10.82–13.35%) in 2011 to 14.93% (95% CI: 13.47–16.51%) in 2018 (*P* for trend < 0.001). RA and PsA remained stable (*P* for trend = 0.220 and 0.849, respectively), while other arthritis rose from 2.03% (95% CI: 1.54–2.67%) in 2011–12 to 3.14% (95% CI: 2.56–3.86%) in 2017–18 (*P* for trend = 0.001). In Whites, Asians, and other races , arthritis and RA prevalence increased significantly (*P* for trend < 0.05). OA and other arthritis rose in Whites and other races (*P* for trend < 0.05), but no significant change occurred in the black population. The prevalence of PsA remained stable across all racial groups, with no statistically significant changes.

**Conclusions:**

In this nationally representative U.S. adult survey spanning 2011 to 2018, we identified a rising prevalence trend in arthritis, OA, and other arthritis, with notable variations among different racial groups.

**Supplementary Information:**

The online version contains supplementary material available at 10.1186/s12889-024-18966-0.

## Introduction

The term “arthritis, ” derived from the Greek language, signifies “joint disease. ” It is characterized by inflammation, acute or chronic, in the joints, often accompanied by pain and structural damage [1]. The common types are osteoarthritis (OA), rheumatoid arthritis (RA), and psoriatic arthritis (PsA) [2, 3]. These conditions may lead to joint discomfort, limited functionality, and decreased mobility, significantly reducing patients’ quality of life [4, 5]. With an aging population and improved survival rates, arthritis incidence and prevalence are on the rise [6–8]. Arthritis, recognized as a leading cause of disability by the World Health Organization, not only causes individual suffering but also poses substantial economic and healthcare burdens on society [6, 9]. Therefore, accurately predicting the incidence and trends of chronic diseases like arthritis is vital for planning clinical and public health strategies, shaping health policies, and allocating resources effectively.

In 2017, the age-standardized prevalence rates of OA [10] and RA [6] globally were 3.75% and 0.25%, respectively, showing increases of 9.3% and 7.4%, respectively, since 1990. Few studies have reported on the prevalence and trends of OA [2, 11, 12], RA [2, 13], and PsA [14, 15] in the U.S., but these results vary. For instance, Park [2] and Hunter [13] have opposing views on the prevalence of RA in the U.S. Park’s study found that the prevalence of RA was 5.9% in 1999−2000, which decreased to 3.8% by 2013−14 [2]; whereas Hunter posits that the prevalence of RA has been on an upward trend from 2004 to 2014 [13]. Regarding PsA, prevalence estimates exhibit considerable variability, ranging from 0.02% to 0.25%, with recent estimates being higher [15]. It is noteworthy that most previous studies have focused primarily on trends in the prevalence of arthritis before 2014, leaving us with limited knowledge about these trends thereafter.

The National Health and Nutrition Examination Survey (NHANES) cycles from 2011 to 2018 oversampled non-Hispanic Asians and provided additional sampling for traditionally oversampled groups, including Hispanic and non-Hispanic Black populations [16]. Prior to the 2011–12 NHANES cycle, data for non-Hispanic Asians were amalgamated with other racial categories in publicly released information. As of 2017, the U.S. Asian population has reached 18.3 million, accounting for 5.7% of the total population, and is expected to reach 36.8 million (9.1%) by 2060 [17]. Despite being the fastest-growing racial group in the U.S. [18], Asians are underrepresented in health research [19], a trend seen in other Western countries. Given the sustained growth of the Asian American population, obtaining comprehensive insights into their health status and trends is imperative.

This study aims to fill a critical data gap by examining the prevalence and trends of arthritis among Asian Americans on a national level, facilitating comparisons with other racial groups. Using NHANES data from 2011 to 2018, we conducted a thorough analysis of arthritis trends in U.S. adults, including OA, RA, PsA, and other arthritis, while accounting for variables like age, sex, and race.

## Methods

### NHANES study population

The NHANES, conducted by the National Center for Health Statistics (NCHS) under the CDC, is a nationally representative survey using a complex, multistage probability sampling design [20]. The survey comprises two main components: interviews and physical examinations. During interviews, participants responded to questions related to demographics, socioeconomic factors, diet, and health. The examinations included medical, dental, and physiological measurements [21]. Since 2011, the NHANES has oversampled non-Hispanic Asians to enhance the statistical precision for this population. Despite a decrease in response rates from 66% in 2011–12 to 47.7% in 2017–18, the CDC has meticulously assessed the data and implemented improved weighting adjustments to minimize potential nonresponse bias [22]. NHANES procedures were approved by the NCHS Institutional Review Board (https://www.cdc.gov/nchs/nhanes/irba98.htm), and written consent was obtained from all adult participants. Additional details on NHANES methods and data acquisition are available on the NHANES website (https://www.cdc.gov/nchs/nhanes/index.htm).

### Arthritis in NHANES

Participants who answered “y es” to the research question “Doctors ever said you had arthritis?” were classified as having arthritis. Further classification into specific diseases was based on responses to the question “Which type of arthritis was it?” with options including “OA or degenerative arthritis, ” “RA, ” “PsA, ” or “Other. ” Our study focused on adults aged 20 years and older who answered questions about arthritis in four NHANES cycles (2011–12 to 2017–18). Among the 22,617 adults who participated in the NHANES from 2011 to 2018, 51 participants lacking arthritis data and 1, 719 participants lacking classification interview data were excluded. Ultimately, we obtained arthritis data from 22,566 adults and arthritis classification data from 20,898 adults.

### Covariates in NHANES

During the interview phase, we employed standardized questionnaires to collect information on age, gender, and race. The NHANES questionnaire is available in two versions: English and Spanish. For participants who were not proficient in English or Spanish, and those with limited English proficiency, an interpreter provided assistance during the interview.  

Racial classifications were self-reported and included Mexican American, Other Hispanic, Non-Hispanic White (referred to as White), Non-Hispanic Black (referred to as Black), Non-Hispanic Asian (encompassing East Asia, Southeast Asia, or the Indian subcontinent, such as Cambodia, China, India, Japan, Korea, Malaysia, Pakistan, the Philippines, Thailand, and Vietnam; referred to as Asian), and Other Race (comprising American Indian, Alaskan Native, Native Hawaiian, Pacific Islander, and individuals with multiple racial backgrounds). Non-Hispanic participants reporting multiple racial backgrounds were categorized as “Other Race” [23, 24].

### Statistical analysis

Following NHANES analysis guidelines, we applied sample weights in the stratified multistage probability design, as recommended by NHANES, to obtain variance estimates. Given the complex nature of the factors influencing arthritis prevalence and for comparison with previous reports from the CDC, we calculated the prevalence of various types of arthritis in the U.S. adult population for each NHANES cycle from 2011– 12 to 2017–18 (expressed as percentages) based on 2000 census data and NHANES recommendations. We conducted stratified analyses by age, sex, and race. Trends over time were explored using logistic regression, treating the survey cycle as a continuous independent variable. Unweighted values and trends were also calculated for sensitivity analysis. Statistical analyses utilized STATA 17.0 and Empower (R) (https://www.empowerstats.net/cn), with statistical significance set at *P* < 0.05.

## Results

This study employed different sample sizes for distinct measurements: the arthritis sample included 22,566 individuals, while samples for OA, RA, PsA, and other arthritis comprised 20,898 individuals. In the overall study sample, the weighted average age was 44.8 years (SE = 0.15); 10,927 were males (weighted 48.08%), and 11,639 were females (weighted 51.92%). There were 3, 030 Mexican Americans (weighted 8.63%), 2, 364 other Hispanic Americans (weighted 6.38%), 8291 Whites (weighted 64.58%), 5, 120 B lacks (weighted 11.45%), 2, 955 Asians (weighted 5.58%), and 806 individuals of  other races (weighted 3.38%). Sample sizes and overall characteristics varied slightly across survey cycles, with detailed descriptions in Supplementary Table S1. Additionally, Supplementary Table S2 presents unweighted sample sizes for adults aged 20 and above in NHANES 2017–18, stratified by sex, age, and race.

### Arthritis

**Fig. 1:**
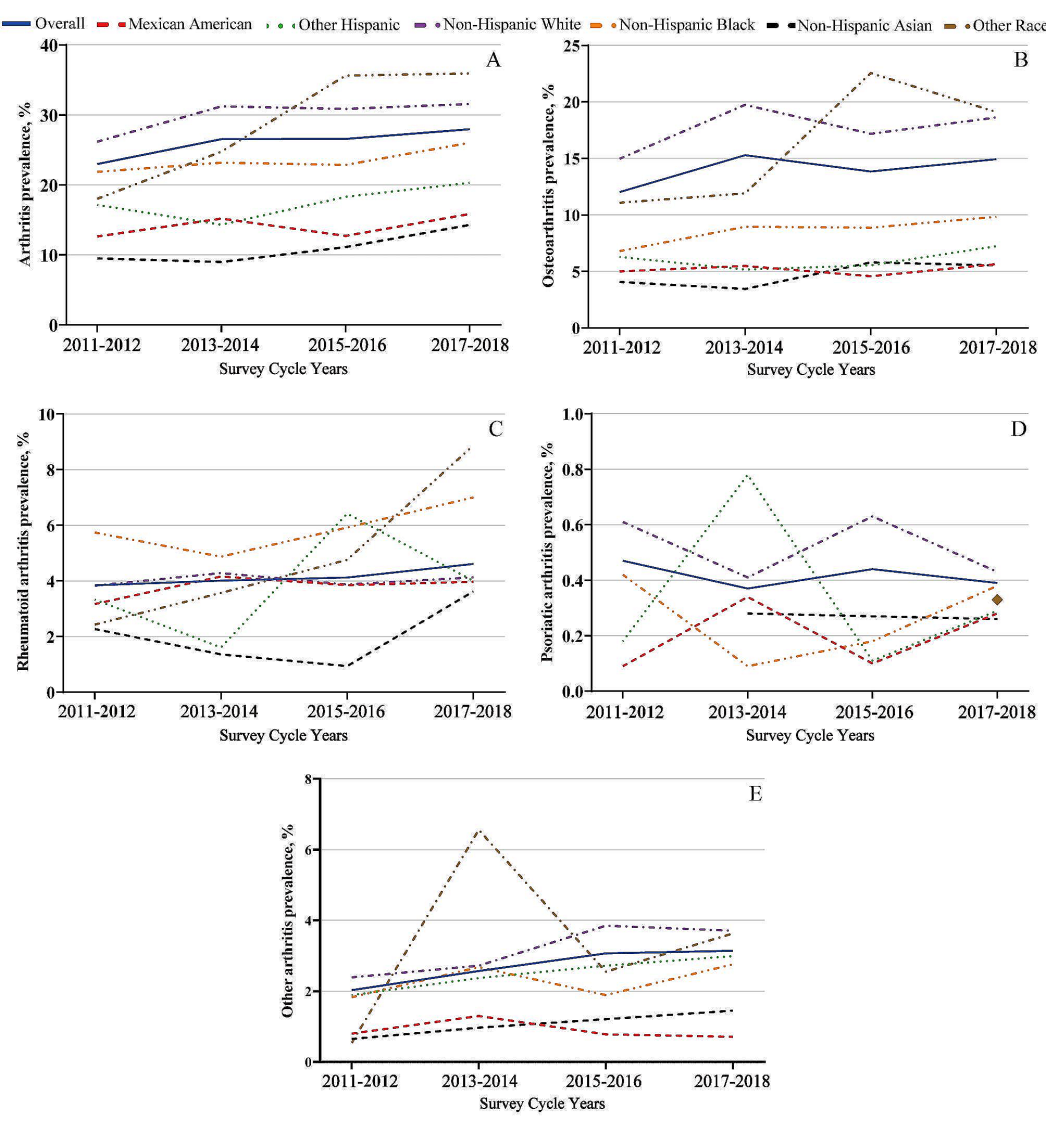
Trends in prevalence of arthritis by race among adults in the United States, 2011–18

Table 1, Fig. 1A, and Supplementary Figure S1A present the arthritis prevalence from 2011 to 2018, along with the estimated prevalence stratified by sex, age group, and race. The overall prevalence significantly increased from 22.98% (95% CI: 21.47–24.55%) in 2011–12 to 27.95% (95% CI: 28.20–30.4%) in 2017–18 (*P* for trend < 0.001). The prevalence among males increased from 18.60% (95% CI: 16.59–20.79%) to 24.72% (95% CI: 22.22–27.40%) (*P* for trend < 0.001), while among females, it increased from 27.00% (95% CI: 24.83–29.30%) to 30.94% (95% CI: 28.55–33.44%) (*P* for trend < 0.001). Stratification by quartile revealed a significant increase in arthritis prevalence among the  51- to 64-year-old age group  (32.58–39.70%, *P* for trend < 0.001) and the  65- to 80-year-old age group (49.92–56.49%, *P* for trend = 0.002).

**Table 1:** Trends in Arthritis Prevalence, National Health and Nutrition Examination Survey, 2011–18 (*n* = 22,566)

Variable	Prevalence, % (95% CI)				
	2011–12	2013–14	2015–16	2017–18	*P* for trend
**Overall**	22.98 (21.47, 24.55)	26.54 (25.11, 28.01)	26.57 (24.97, 28.23)	27.95 (26.20, 29.76)	**< 0.001**
**All participants**					
Mexican American	12.63 (10.15, 15.63)	15.19 (12.94, 17.75)	12.71 (10.89, 14.79)	15.85 (13.20, 18.92)	0.160
Other Hispanic	17.16 (14.40, 20.33)	14.31 (11.60, 17.54)	18.29 (15.72, 21.17)	20.30 (16.79, 24.34)	0.062
Non-Hispanic White	26.18 (24.03, 28.46)	31.23 (29.20, 33.33)	30.87 (28.52, 33.32)	31.57 (28.99, 34.27)	**< 0.001**
Non-Hispanic Black	21.87 (19.81, 24.07)	23.18 (1.25, 20.82)	22.86 (20.56, 25.34)	26.02 (23.55, 28.64)	0.076
Non-Hispanic Asian	9.50 (7.63, 11.78)	8.98 (7.00, 11.45)	11.11 (8.95, 13.72)	14.28 (11.98, 16.94)	**0.004**
Other Race	18.02 (10.99, 28.14)	24.75 (17.41, 33.92)	35.63 (27.15, 45.12)	35.93 (26.87, 46.11)	**< 0.001**
**Age (yrs)**					
20–34	3.88 (2.75, 5.44)	5.21 (3.94, 6.85)	3.93 (2.86, 5.39)	5.00 (3.67, 6.77)	0.213
35–50	13.61 (11.42, 16.14)	15.28 (13.24, 17.57)	15.59 (13.29, 18.20)	15.51 (12.97, 18.45)	0.387
51–64	32.58 (28.80, 36.60)	38.42 (34.96, 41.99)	37.72 (33.92, 41.69)	39.70 (35.47, 44.08)	**< 0.001**
65–80	49.92 (46.17, 53.68)	55.44 (52.09, 58.74)	55.29 (51.54, 58.99)	56.49 (52.74, 60.17)	**0.002**
Trend of affected age	60.26 ± 13.99	59.62 ± 13.80	60.45 ± 13.53	60.56 ± 13.83	0.227
**Male participants**					
Mexican American	10.25 (7.21, 14.37)	11.04 (8.46, 14.27)	10.28 (8.00, 13.12)	12.17 (8.80, 16.58)	0.820
Other Hispanic	11.67 (8.42, 15.95)	10.67 (7.33, 15.28)	13.34 (10.11, 17.41)	15.79 (11.21, 21.79)	0.324
Non-Hispanic White	21.97 (19.11, 25.12)	24.78 (22.05, 27.73)	25.54 (22.41, 28.94)	28.43 (24.78, 32.39)	**0.009**
Non-Hispanic Black	15.29 (12.90, 18.02)	16.78 (13.93, 20.07)	15.86 (13.12, 19.06)	22.21 (18.88, 25.95)	**0.004**
Non-Hispanic Asian	5.84 (3.90, 8.65)	4.69 (2.85, 7.64)	6.14 (4.04, 9.24)	11.95 (8.93, 15.80)	**< 0.001**
Other Race	14.38 (6.35, 29.38)	23.68 (13.59, 37.97)	33.89 (22.93, 46.89)	33.65 (20.94, 49.25)	**0.009**
All male participants	18.60 (16.59, 20.79)	20.76 (18.84, 22.82)	21.54 (19.40, 23.84)	24.72 (22.22, 27.40)	**< 0.001**
Age (yrs)	59.82 ± 13.48	58.81 ± 13.87	59.87 ± 13.40	59.46 ± 13.34	0.526
**Female participants**					
Mexican American	15.29 (11.54, 19.99)	19.76 (16.19, 23.89)	15.14 (12.44, 18.31)	19.77 (15.89, 24.33)	0.135
Other Hispanic	21.96 (17.84, 26.73)	17.36 (13.47, 22.09)	23.03 (19.29, 27.25)	24.26 (19.31, 30.02)	0.175
Non-Hispanic White	30.11 (27.00, 33.42)	37.24 (34.35, 40.23)	35.90 (32.52, 39.42)	34.48 (30.91, 38.24)	**0.004**
Non-Hispanic Black	27.09 (24.02, 30.40)	28.39 (24.89, 32.16)	28.47 (25.08, 32.11)	29.17 (25.70, 32.91)	0.865
Non-Hispanic Asian	12.65 (9.75, 16.26)	12.61 (9.48, 16.59)	15.46 (12.04, 19.65)	16.25 (13.02, 20.10)	0.339
Other Race	21.56 (11.44, 36.89)	25.91 (16.42, 38.36)	37.28 (25.19, 51.20)	38.68 (26.97, 51.86)	**0.033**
All female participants	27.00 (24.83, 29.30)	31.89 (29.85, 33.99)	31.23 (28.94, 33.62)	30.94 (28.55, 33.44)	**< 0.001**
Age (yrs)	60.54 ± 14.30	60.11 ± 13.73	60.82 ± 13.60	61.38 ± 14.12	0.248

In addition, we observed that the prevalence of arthritis among females was consistently higher than that among males across all years (Supplementary Figure S1**A**). The rates increased significantly in the White, Asian, and other racial groups (*P* for trend < 0.001, 0.004, < 0.001, respectively). The increase observed in Black and Asian populations was primarily among males, while increases in W hite and other racial groups were observed in both sexes. Supplementary Table S3 presents the unweighted arthritis prevalence and trends, with notable differences in overall prevalence observed in the 20- to 34-year-old and 25- to 50-year-old age groups , followed by W hite women.

### Osteoarthritis

In the overall population, the weighted prevalence of OA was 12.02% (95% CI: 10.82–13.35%), 15.29% (95% CI: 14.06–16.60%), 13.84% (95% CI: 12.53–15.27%) and 14.93% (95% CI: 13.47–16.51%) from 2011– 12 to 2017–18, respectively (*P* trend for < 0.001) (Table 2 and Fig. 1B). An increase in prevalence was primarily observed in White individuals (14.97–18.63%, *P* for trend < 0.001) and other races (11.07–19.10%, *P* for trend < 0.009). When stratified by age, we observed changes consistent with those of arthritis. H owever, in the 20- to 34-year-old age group, there was a decrease from 1.86% (95% CI: 1.12–3.10%) in 2011–12 to 0.99% (95% CI: 0.59–1.64%) in 2017–18 (*P* for trend = 0.007). Increases in prevalence were observed in both sexes (Table 2 and Supplementary Figure S1B), with a notable increase in the other racial group of male from 3.96% (95% CI: 1.56–9.72%) in 2011–12 to 16.89% (95% CI: 8.36–31.14%) in 2017–18, while growth in females was primarily observed in the W hite population (*P* for trend = 0.009). Supplementary Table S4 displays the unweighted prevalence and trends of OA.

**Table 2:** Trends in Osteoarthritis Prevalence, National Health and Nutrition Examination Survey, 2011–18 (*n* = 20,898)

Variable	Prevalence, % (95% CI)				
	2011–12	2013–14	2015–16	2017–18	*P* for trend
**Overall**	12.02 (10.82, 13.35)	15.29 (14.06, 16.60)	13.84 (12.53, 15.27)	14.93 (13.47, 16.51)	**< 0.001**
**All participants**					
Mexican American	5.00 (3.39, 7.30)	5.48 (4.13, 7.22)	4.57 (3.48, 5.96)	5.64 (4.03, 7.84)	0.790
Other Hispanic	6.28 (4.66, 8.42)	5.16 (3.61, 7.33)	5.54 (4.17, 7.33)	7.23 (5.14, 10.09)	0.493
Non-Hispanic White	14.97 (13.23, 16.90)	19.74 (17.96, 21.65)	17.18 (15.24, 19.32)	18.63 (16.44, 21.03)	**< 0.001**
Non-Hispanic Black	6.80 (5.55, 8.31)	8.95 (7.35, 10.85)	8.87 (7.32, 10.70)	9.84 (8.16, 11.81)	0.067
Non-Hispanic Asian	4.07 (2.89, 5.71)	3.45 (2.29, 5.15)	5.86 (4.31, 7.91)	5.53 (4.08, 7.47)	0.101
Other Race	11.07 (5.72, 20.33)	11.91 (6.65, 20.40)	22.56 (15.25, 32.05)	19.10 (12.05, 28.92)	**0.009**
**Age (yrs)**					
20–34	1.86 (1.12, 3.10)	2.57 (1.70, 3.89)	1.25 (0.66, 2.37)	0.99 (0.59, 1.64)	**0.007**
35–50	4.80 (3.57, 6.41)	7.01 (5.55, 8.82)	5.84 (4.45, 7.62)	7.30 (5.35, 9.89)	**0.021**
51–64	17.25 (14.11, 20.92)	22.40 (19.29, 25.85)	18.90 (15.73, 22.52)	22.10 (18.43, 26.26)	**0.002**
65–80	32.31 (28.61, 36.26)	37.45 (33.96, 41.07)	36.19 (32.25, 40.33)	35.12 (31.28, 39.17)	0.065
Trend of affected age	60.02 ± 13.83	59.53 ± 13.73	60.72 ± 13.43	60.79 ± 13.44	0.084
**Male participants**					
Mexican American	4.14 (2.18, 7.71)	4.09 (2.59, 6.40)	3.16 (1.92, 5.16)	3.15 (1.48, 6.56)	0.823
Other Hispanic	5.30 (3.23, 8.58)	2.81 (1.41, 5.54)	4.54 (2.78, 7.31)	3.39 (1.57, 7.17)	0.520
Non-Hispanic White	11.34 (9.15, 13.97)	14.64 (12.37, 17.25)	11.80 (9.50, 14.57)	14.09 (11.30, 17.42)	0.080
Non-Hispanic Black	4.55 (3.20, 6.43)	6.10 (4.32, 8.54)	5.14 (3.52, 7.44)	8.10 (5.91, 11.00)	0.062
Non-Hispanic Asian	2.74 (1.47, 5.04)	2.96 (1.55, 5.56)	3.75 (2.16, 6.45)	2.52 (1.31, 4.81)	0.778
Other Race	3.96 (1.56, 9.72)	15.03 (6.92, 29.63)	22.76 (12.68, 37.43)	16.89 (8.36, 31.14)	**0.013**
All male participants	8.89 (7.47, 10.77)	11.43 (9.86, 13.23)	9.70 (8.12, 11.55)	11.14 (9.27, 13.34)	**0.011**
Age (yrs)	60.69 ± 13.30	59.64 ± 13.27	61.21 ± 12.99	60.59 ± 13.11	0.614
**Female participants**					
Mexican American	5.97 (3.70, 9.49)	7.06 (4.94, 10.00)	5.99 (4.36, 8.19)	8.34 (5.81, 11.82)	0.545
Other Hispanic	7.20 (4.95, 10.35)	7.19 (4.73, 10.77)	6.53 (4.63, 9.15)	10.63 (7.28, 15.28)	0.214
Non-Hispanic White	18.39 (15.81, 21.29)	24.57 (21.95, 27.39)	22.20 (19.24, 25.47)	22.76 (19.57, 26.29)	**0.009**
Non-Hispanic Black	8.66 (6.76, 11.04)	11.38 (8.96, 14.35)	11.96 (9.59, 14.82)	11.31 (8.97, 14.18)	0.268
Non-Hispanic Asian	5.25 (3.47, 7.87)	3.88 (2.28, 6.52)	7.79 (5.40, 11.13)	8.10 (5.75, 11.29)	0.055
Other Race	17.40 (8.15, 33.34)	8.47 (3.76, 17.99)	22.36 (13.01, 35.67)	21.83 (11.79, 36.83)	0.078
All female participants	14.86 (13.07, 16.85)	18.95 (17.14, 20.89)	17.70 (15.71, 19.87)	18.42 (16.29, 20.76)	**< 0.001**
Age (yrs)	62.83 ± 13.67	61.51 ± 13.14	62.98 ± 12.39	63.13 ± 12.62	0.223

### Rheumatoid arthritis

The prevalence of RA remained stable from 2011 to 2018, with rates of 3.84% (95% CI: 3.20–4.61%), 4.01% (95% CI: 3.42–4.69%), 4.12% (95% CI: 3.50–4.85%), and 4.61% (95% CI: 3.88–5.48%) (*P* for trend = 0.220) (Table 3; Fig. 1C), with no statistically significant differences observed among age groups. When stratified by race, while B lack individuals had the highest prevalence, the rate remained stable (*P* for trend = 0.181) (Table 3). However, an increasing trend in RA prevalence was observed in other Hispanic (*P* for trend < 0.001), Asian (*P* for trend = 0.001), and other racial groups (*P* for trend = 0.023); among these, an increase in prevalence among Asians (*P* for trend < 0.001) and other racial groups (*P* for trend < 0.001) was mainly driven by males, while in the other Hispanic group, the increase was primarily seen in females (*P* for trend = 0.006). Gender-stratified analysis revealed an increase in prevalence only in males (Table 3 and Supplementary Figure S1C), with a gradual increase in age at onset observed in males (*P* for trend = 0.024), while no such difference was observed among females (eFig. 1C). Supplementary Table S5 presents the unweighted prevalence and trends of RA.

**Table 3:** Trends in Rheumatoid Arthritis Prevalence, National Health and Nutrition Examination Survey, 2011–18 (*n* = 20,898)

Variable	Prevalence, % (95% CI)				
	2011–12	2013–14	2015–16	2017–18	*P* for trend
**Overall**	3.84 (3.20, 4.61)	4.01 (3.42, 4.69)	4.12 (3.50, 4.85)	4.61 (3.88, 5.48)	0.220
**All participants**					
Mexican American	3.17 (2.03, 4.92)	4.16 (3.04, 5.68)	3.85 (2.82, 5.23)	3.97 (2.64, 5.92)	0.803
Other Hispanic	3.33 (2.16, 5.11)	1.61 (0.89, 2.90)	6.42 (4.82, 8.50)	4.00 (2.66, 5.98)	**< 0.001**
Non-Hispanic White	3.83 (2.95, 4.97)	4.28 (3.46, 5.27)	3.87 (3.00, 4.98)	4.13 (3.20, 5.31)	0.880
Non-Hispanic Black	5.74 (4.64, 7.08)	4.87 (3.73, 6.32)	5.92 (4.69, 7.44)	7.00 (5.71, 8.55)	0.181
Non-Hispanic Asian	2.27 (1.41, 3.61)	1.36 (0.69, 2.67)	0.94 (0.42, 2.12)	3.62 (2.48, 5.26)	**0.001**
Other Race	2.43 (0.94, 6.13)	3.57 (1.43, 8.63)	4.76 (2.65, 8.38)	8.87 (3.45, 20.92)	**0.023**
**Age (yrs)**					
20–34	0.52 (0.18, 1.48)	1.06 (0.55, 2.02)	0.88 (0.47, 1.64)	1.10 (0.56, 2.17)	0.386
35–50	3.46 (2.40, 4.97)	2.50 (1.74, 3.59)	2.39 (1.68, 3.40)	2.47 (1.63, 3.72)	0.248
51–64	5.90 (4.23, 8.19)	6.26 (4.76, 8.21)	6.61 (4.98, 8.73)	7.74 (5.65, 10.50)	0.269
65–80	6.64 (5.20, 8.45)	7.69 (6.09, 9.66)	8.06 (6.26, 10.31)	8.33 (6.69, 10.32)	0.438
Trend of affected age	56.94 ± 13.86	57.61 ± 14.79	59.32 ± 14.34	58.64 ± 13.69	0.208
**Male participants**					
Mexican American	2.65 (1.36, 5.12)	3.11 (1.88, 5.10)	3.33 (2.08, 5.31)	3.23 (1.57, 6.51)	0.962
Other Hispanic	1.22 (0.51, 2.88)	1.34 (0.47, 3.75)	4.36 (2.56, 7.34)	3.11 (1.62, 5.89)	0.082
Non-Hispanic White	3.41 (2.31, 5.00)	3.22 (2.25, 4.60)	3.46 (2.34, 5.09)	4.57 (3.15, 6.59)	0.393
Non-Hispanic Black	3.63 (2.55, 5.14)	3.91 (2.60, 5.83)	4.05 (2.76, 5.90)	5.69 (4.16, 7.74)	0.306
Non-Hispanic Asian	1.33 (0.59, 2.98)	0.32 (0.04, 2.21)	1.16 (0.43, 3.09)	4.13 (2.37, 7.09)	**< 0.001**
Other Race	1.16 (0.26, 5.00)	-	3.73 (1.58, 8.55)	11.17 (3.00, 33.82)	**< 0.001**
All male participants	3.06 (2.27, 4.11)	2.93 (2.23, 3.84)	3.44 (2.64, 4.48)	4.79 (3.61, 6.32)	**0.001**
Age (yrs)	56.10 ± 14.11	55.69 ± 14.70	60.95 ± 13.38	57.66 ± 13.37	**0.024**
**Female participants**					
Mexican American	3.75 (2.05, 6.76)	5.37 (3.58, 7.98)	4.37 (2.89, 6.55)	4.77 (2.98, 7.56)	0.784
Other Hispanic	5.29 (3.24, 8.53)	1.85 (0.91, 3.73)	8.46 (6.04, 11.73)	4.79 (2.84, 7.98)	**0.006**
Non-Hispanic White	4.23 (2.96, 6.01)	5.28 (4.07, 6.82)	4.26 (3.05, 5.92)	3.72 (2.64, 5.22)	0.394
Non-Hispanic Black	7.48 (5.75, 9.66)	5.68 (4.02, 7.98)	7.47 (5.59, 9.91)	8.11 (6.22, 10.51)	0.410
Non-Hispanic Asian	3.09 (1.75,5.43)	2.28 (1.11, 4.66)	0.75 (0.19, 2.94)	3.19 (1.91, 5.29)	0.095
Other Race	3.55 (1.12, 10.70)	7.49 (3.02, 17.39)	5.75 (2.62, 12.15)	6.03 (2.86, 12.30)	0.786
All female participants	4.57 (3.62, 5.75)	5.02 (4.14, 6.08)	4.76 (3.87, 5.84)	4.46 (3.64, 5.45)	0.778
Age (yrs)	57.46 ± 13.67	58.67 ± 14.73	58.22 ± 14.86	59.61 ± 13.95	0.599

### Psoriatic arthritis

Over the period from 2011 to 2018, the prevalence of PsA among adults in the U.S. remained stable (*P* for trend = 0.849), both in the overall population and when stratified by sex, age, and race (*P* for trend > 0.05) (Table 4; Fig. 1D; and Supplementary Figure S1D). Supplementary Table S6 provides a detailed overview of the unweighted prevalence and trends of PsA, showing no significant differences.

**Table 4:** Trends in Psoriatic Arthritis Prevalence, National Health and Nutrition Examination Survey, 2011–18 (*n* = 20,898)

Variable	Prevalence, % (95% CI)				
	2011–12	2013–14	2015–16	2017–18	*P* for trend
**Overall**	0.47 (0.24, 0.93)	0.37 (0.22, 0.64)	0.44 (0.23, 0.83)	0.39 (0.17, 0.86)	0.849
**All participants**					
Mexican American	0.09 (0.01, 0.63)	0.34 (0.10, 1.22)	0.10 (0.03, 0.42)	0.28 (0.07, 1.09)	0.653
Other Hispanic	0.18 (0.05, 0.74)	0.78 (0.25, 2.46)	0.11 (0.03, 0.45)	0.29 (0.05, 1.59)	0.243
Non-Hispanic White	0.61 (0.28, 1.34)	0.41 (0.20, 0.83)	0.63 (0.31, 1.26)	0.43 (0.14, 1.32)	0.692
Non-Hispanic Black	0.42 (0.19, 0.91)	0.09 (0.01, 0.61)	0.18 (0.04, 0.71)	0.38 (0.16, 0.89)	0.344
Non-Hispanic Asian	-	0.28 (0.07, 1.11)	-	0.26 (0.07, 1.07)	0.281
Other Race	-	-	-	0.33 (0.05, 2.29)	0.655
**Age (yrs)**					
20–34	0.03 (0.00, 0.25)	0.05 (0.01, 0.39)	-	0.12 (0.02, 0.83)	0.598
35–50	0.87 (0.35, 2.16)	0.29 (0.11, 0.76)	0.51 (0.22, 1.18)	0.26 (0.11, 0.60)	0.067
51–64	0.64 (0.17, 2.36)	0.53 (0.21, 1.31)	0.55 (0.13, 2.29)	0.92 (0.26, 3.19)	0.081
65–80	0.24 (0.10, 0.59)	0.76 (0.28, 2.01)	0.80 (0.28, 2.25)	0.25 (0.12, 0.52)	0.849
Trend of affected age	51.69 ± 9.48	57.58 ± 14.58	56.97 ± 12.54	54.07 ± 11.55	0.386
**Male participants**					
Mexican American	0.17 (0.02, 1.19)	0.35 (0.05, 2.47)	0.21 (0.05, 0.84)	0.54 (0.14, 2.08)	0.822
Other Hispanic	0.21 (0.03, 1.48)	0.20 (0.03, 1.41)	0.23 (0.06, 0.91)	-	0.902
Non-Hispanic White	0.57 (0.18, 1.76)	0.37 (0.13, 1.08)	0.49 (0.22, 1.13)	0.64 (0.14, 2.91)	0.861
Non-Hispanic Black	0.26 (0.06, 1.12)	-	-	0.25 (0.08, 0.78)	0.412
Non-Hispanic Asian	-	-	-	0.24 (0.03, 1.70)	0.480
Other Race	-	-	-	-	-
All male participants	0.44 (0.16, 1.18)	0.29 (0.11, 0.74)	0.35 (0.16, 0.74)	0.49 (0.14, 1.70)	0.663
Age (yrs)	49.13 ± 7.20	53.49 ± 14.41	52.55 ± 15.22	58.62 ± 5.33	0.245
**Female participants**					
Mexican American	-	0.33 (0.07, 1.47)	-	-	0.318
Other Hispanic	0.16 (0.02, 1.13)	1.29 (0.36, 4.54)	-	0.54 (0.08, 2.96)	0.128
Non-Hispanic White	0.65 (0.22, 1.91)	0.45 (0.18, 1.14)	0.75 (0.28, 2.04)	0.24 (0.08, 0.68)	0.411
Non-Hispanic Black	0.56 (0.22, 1.38)	0.16 (0.02, 1.12)	0.33 (0.08, 1.30)	0.49 (0.16, 1.50)	0.695
Non-Hispanic Asian	-	0.53 (0.13, 2.08)	-	0.28 (0.04, 1.99)	0.340
Other Race	-	-	-	0.72 (0.10, 5.02)	0.617
All female participants	0.51 (0.20, 1.28)	0.45 (0.23, 0.87)	0.52 (0.20, 1.31)	0.29 (0.15, 0.57)	0.574
Age (yrs)	53.75 ± 10.52	60.10 ± 14.11	59.74 ± 9.52	47.05 ± 14.65	0.122

### Other Arthritis

The overall prevalence of other arthritis increased from 2.03% (95% CI: 1.54–2.67%) in 2011–12 to 3.14% (95% CI: 2.56–3.86%) in 2017–18 (*P* for trend = 0.001) (Table 5; Fig. 1E). However, this increasing trend was significant only for White individuals (*P* for trend = 0.021) and individuals from other racial backgrounds (*P * for trend = 0.033). Age-stratified analysis revealed that growth was primarily concentrated in the 35- to 50-year-old age group  (*P* for trend = 0.009) and the  65- to 80-year-old age group (*P* for trend = 0.002). Gender-stratified analysis indicated an increase in prevalence in both sexes, but it was more pronounced in the female population (*P* for trend = 0.022), while the male population did not show significant differences (*P* for trend = 0.091) (Table 5 and Supplementary Figure S1**E**). Supplementary Table S7 provides unweighted prevalence and trends for other arthritis, demonstrating opposite trends between male and female populations in weighted data.

**Table 5:** Trends in Other Arthritis Prevalence, National Health and Nutrition Examination Survey, 2011–18 (*n* = 20,898)

Variable	Prevalence, % (95% CI)				
	2011–12	2013–14	2015–16	2017–18	*P* for trend
**Overall**	2.03 (1.54, 2.67)	2.57 (2.08, 3.18)	3.07 (2.45, 3.83)	3.14 (2.56, 3.86)	**0.001**
**All participants**					
Mexican American	0.80 (0.31, 2.06)	1.30 (0.73, 2.30)	0.78 (0.42, 1.46)	0.71 (0.33, 1.51)	0.618
Other Hispanic	1.89 (1.05, 3.40)	2.37 (1.26, 4.43)	2.72 (1.77, 4.15)	2.99 (1.56, 5.65)	0.674
Non-Hispanic White	2.39 (1.70, 3.36)	2.72 (2.05, 3.59)	3.85 (2.92, 5.05)	3.71 (2.85, 4.81)	**0.021**
Non-Hispanic Black	1.83 (1.24, 2.69)	2.68 (1.85, 3.86)	1.89 (1.23, 2.87)	2.76 (1.94, 3.92)	0.273
Non-Hispanic Asian	0.65 (0.26, 1.58)	0.97 (0.45, 2.09)	1.21 (0.60, 2.44)	1.45 (0.82, 2.55)	0.511
Other Race	0.54 (0.08, 3.74)	6.56 (2.88, 14.22)	2.55 (0.99, 6.42)	3.63 (1.60, 8.03)	**0.033**
**Age (yrs)**					
20–34	0.48 (0.24, 0.95)	0.54 (0.26, 1.14)	0.50 (0.22, 1.13)	0.82 (0.40, 1.68)	0.626
35–50	1.72 (0.94, 3.12)	2.42 (1.63, 3.56)	3.72 (2.49, 5.53)	2.84 (1.82, 4.42)	**0.009**
51–64	3.36 (2.11, 5.32)	4.26 (2.96, 6.12)	4.13 (2.78, 6.10)	3.74 (2.56, 5.43)	0.646
65–80	3.05 (1.98, 4.68)	3.55 (2.47, 5.09)	4.41 (2.94, 6.57)	5.95 (4.34, 8.11)	**0.002**
Trend of affected age	55.69 ± 13.28	55.08 ± 13.54	55.65 ± 14.22	57.98 ± 14.82	0.280
**Male participants**					
Mexican American	0.17 (0.02, 1.17)	1.13 (0.51, 2.47)	0.83 (0.34, 2.03)	0.58 (0.22, 1.50)	0.493
Other Hispanic	1.91 (0.78, 4.65)	3.33 (1.51, 7.16)	2.10 (0.97, 4.51)	3.54 (1.33, 9.14)	0.578
Non-Hispanic White	2.37 (1.40, 3.98)	2.68 (1.78, 4.01)	3.50 (2.30, 5.31)	3.47 (2.33, 5.12)	0.372
Non-Hispanic Black	1.09 (0.54, 2.20)	2.12 (1.15, 3.87)	1.80 (0.97, 3.31)	2.95 (1.74, 4.96)	0.161
Non-Hispanic Asian	0.77 (0.24, 2.46)	0.55 (0.14, 2.16)	0.52 (0.13, 2.08)	1.88 (0.91, 3.86)	0.184
Other Race	1.14 (0.16, 7.76)	6.69 (2.43, 17.07)	2.23 (0.77, 6.31)	2.03 (0.60, 6.64)	0.139
All male participants	1.90 (1.22, 2.96)	2.51 (1.84, 3.41)	2.75 (1.94, 3.88)	2.97 (3.19, 4.02)	0.091
Age (yrs)	56.73 ± 11.38	56.38 ± 14.08	52.63 ± 12.53	58.38 ± 13.25	0.066
**Female participants**					
Mexican American	1.52 (0.54, 4.21)	1.49 (0.64, 3.40)	0.73 (0.31, 1.72)	0.85 (0.28, 2.56)	0.647
Other Hispanic	1.87 (0.86, 4.04)	1.55 (0.53, 4.42)	3.33 (2.02, 5.45)	2.50 (1.14, 5.43)	0.514
Non-Hispanic White	2.41 (1.53, 3.75)	2.75 (1.87, 4.03)	4.17 (2.90, 5.95)	3.92 (2.76, 5.55)	0.080
Non-Hispanic Black	2.44 (1.53, 3.85)	3.16 (1.99, 4.98)	1.96 (1.09, 3.49)	2.60 (1.62, 4.14)	0.622
Non-Hispanic Asian	0.54 (0.14, 2.15)	1.35 (0.54, 3.35)	1.85 (0.82, 4.10)	1.09 (0.44, 2.67)	0.457
Other Race	-	6.41 (1.67, 21.64)	2.86 (0.67, 11.47)	5.61 (1.97, 14.95)	0.148
All female participants	2.15 (1.52, 3.03)	2.64 (1.97, 3.52)	3.36 (2.51, 4.50)	3.30 (2.50, 4.36)	**0.022**
Age (yrs)	54.84 ± 14.61	53.91 ± 12.92	57.95 ± 14.99	57.65 ± 16.00	0.245

## Discussion

This study, using nationally representative data encompassing diverse racial backgrounds, offers detailed estimates of the national trends in various types of arthritis among U.S. adults from 2011–12 to 2017–18. We explore these trends across age, sex, and racial groups. Overall, the prevalence of arthritis in U.S. adults remains relatively high and is on the rise, although significant differences exist among different sexes, age groups, and racial groups . The White population shows an increasing trend in arthritis, OA, and other types of arthritis, while the Asian population exhibits an increase in the prevalence of arthritis and RA. The other Hispanic group only showed an increase in RA prevalence. In contrast, Black and Mexican American individuals demonstrate relatively stable prevalence rates across various types of arthritis. Other racial groups are the only ones showing an increase in all types of arthritis, except for PsA.

In the U.S., doctor-diagnosed arthritis is a prevalent chronic condition [25, 26] and a major cause of disability [27], contributing to approximately $81 billion in annual direct medical expenses related to arthritis [28]. Annually, approximately one million knee and hip replacements are performed, 99% of which are attributed to pain and functional limitations caused by arthritis [29]. The aging population is a driving factor in predicting the prevalence of arthritis  and its associated impacts [30]. This study revealed an arthritis prevalence of 56.49% in the population aged 65 and older, which was significantly greater than that in the population younger than 65 years. According to the U.S. Census Bureau, by 2030, one-fifth of U.S. adults will be aged 65 or older [31], and arthritis prevalence is expected to continue to increase. Hootman et al. reported that between 2010 and 2012, 52.5 million adults (22.7% of all adults) had doctor-diagnosed arthritis. P rojections indicate that by 2040, the number of U.S. adults with doctor-diagnosed arthritis will reach 78.4 million (25.9% of all adults) [11]. Our study revealed that in 2011–12, the prevalence of arthritis among U.S. adults was approximately 22.98%, which is consistent with previous estimates. However, by 2013–14, the prevalence had exceeded the 25.9% predicted by Hootman et al., reaching 26.54%. This further substantiates the ongoing trend of increasing arthritis prevalence in the future.

OA is the most prevalent joint disease in developed countries and primarily impacts the knee or hip joints [32–34]. The etiology of OA is diverse, stemming from the combined effects of various factors [32, 35]. Research on different races revealed that in the U.S., the prevalence of OA is notably high among W hite individuals, reaching 18.63%, while Asians exhibit a lower rate of 5.53%. When considering the non-modifiable factors of OA, age and sex were considered to be the strongest predictors. Specifically, females are more susceptible to OA than males [35–37], a finding substantiated in our study (males: 11.14%, females: 18.42%). The influence of age may closely correlate with changes in joint biomechanics [33]. In our investigation, the prevalence of OA in the 20- to 34-year-old age group in 2017–18 was 0.99%, which significantly increased to 35.12% in the 65- to 80-year-old age group. Furthermore, obesity is considered a highly influential modifiable risk factor for the development of OA [32, 38]. A meta-analysis revealed that the likelihood of knee OA in individuals classified as obese or overweight is nearly three times greater than that in individuals with normal body weight [38]. In terms of geographical prevalence, the rate we observed, at 14.93%, is consistent with the range reported across Europe, which spans from 10 to 17% [39]. It also closely matches the prevalence found in prior research conducted in the United States, at 13.9% [34]. However, when compared to South America, our figure is considerably higher, given that estimates there are significantly lower, ranging from 2 to 4% [39]. Additionally, our observed rate is notably lower than those in Asian, African, and Middle Eastern regions, where the prevalence is reported to be higher, specifically within the ranges of 16–23% [39], 17–25% [39], and 17–29% [40, 41], respectively. We observed a 24.21% increase in OA prevalence from 2011 to 2018, further confirming existing epidemiological evidence indicating a rising trend in OA prevalence [10, 42]. The increase in OA prevalence may reflect population aging, an increase in factors contributing to OA risk, and heightened awareness of OA.

RA is an autoimmune disease characterized by joint inflammation and the potential for destructive bone erosion [43], affecting approximately 1% of the global population [44, 45]. RA is considered a multifactorial disease influenced by various genetic and environmental factors [46, 47], contributing to variations in prevalence both between and within countries [48]. Among RA patients, the prevalence of work-related disabilities related to RA is estimated to be approximately 35% [49]. Multiple studies on the trends in RA incidence in the U.S. have reported inconsistent results [2, 13]. Approximately 1.3 million American adults, constituting 0.6–1% of the adult population, are affected by RA [34, 50]. However, our research revealed that the prevalence of RA ranged from 3.84% to 4.61% between 2011 and 2018. Park et al. reported a decreasing trend in the incidence of RA among American adults from 1999 to 2014 [2], while Hunter et al. argued that during the same period, the incidence of RA in the U.S. seemed to increase [13]. In addition, these studies only evaluated the trend of RA before 2014, and the subsequent trends are still unknown. The most recent study based on the NHANES reported the prevalence of RA for the years 2017–18, revealing an increase in the incidence among males and a decrease among females [51], a trend validated by our research. Regarding race, the results showed an increasing trend in prevalence among the other Hispanic (*P* for trend < 0.001), Asian (*P* for trend = 0.001), and other racial groups (*P* for trend = 0.023).

PsA is a chronic inflammatory musculoskeletal disease that is usually negative for rheumatoid factors in the blood and is associated with psoriasis. The general population’s prevalence ranges from 0.02% to 0.42%, with 13.8–30% among psoriasis patients [52]. Global PsA prevalence studies encompass multiple countries, estimating that the prevalence in the U.S. is between 0.06% and 0.25%, while Sweden and Norway exhibit rates ranging from 0.02% to 0.67% [53, 54]. Reports on PsA prevalence in South America and Asia are limited, suggesting lower rates in these regions (e.g., China at 0.02%) [55, 56]. While research on the prevalence of PsA in the general population is relatively limited, a recent meta-analysis incorporating 28 studies indicated a global prevalence of approximately 0.13% [57]. Our study estimates that the prevalence of PsA among U.S. adults in 2017–18 was approximately 0.39%, surpassing the global average. The occurrence of this phenomenon can be attributed to several factors: firstly, racial/ethnic differences, with the prevalence rate of the disease typically being higher in non-Hispanic Whites than in Blacks [58]; secondly, geographical disparities, with the prevalence rate often being lower in regions with abundant sunshine [59]; and lastly, differences in the methods of diagnosis and reporting, which can also influence the statistical reporting of prevalence rates [60].

Furthermore, other arthritis in the study may include joint diseases with low prevalence, such as reactive arthritis, Kaschin-Beck disease, or hemophiliac arthritis. The results indicate a recent increase in the incidence of these types of arthritis (*P* for trend = 0.001), predominantly observed in White individuals  and other races, as well as among individuals aged 35 to 50 years and 65 to 80 years . Due to the lack of specific subgrouping for these types of arthritis in our study, the exact prevalence rates remain unknown. Further research is needed to elucidate the epidemiological patterns of these patients with low-prevalence arthritis conditions .

### Strengths and limitations

A major strength of this study lies in the utilization of NHANES data, which provides an opportunity to assess the nationwide prevalence and trends of arthritis, including OA, RA, PsA, and other forms of arthritis. Additionally, our study employs a sufficiently large sample size, allowing for differentiation among non-Hispanic Asians and other racial groups, thereby revealing a distinctive pattern of arthritis prevalence among Asian Americans—a pattern that has been overlooked in prior research.

Similarly, this study has several limitations. First, we relied on self-reported physician-diagnosed arthritis data, introducing the possibility of participant recall and self-reporting biases. Second, although the NHANES remains a leading national survey with a relatively high response rate, similar to many other national face-to-face surveys [61], response rates have gradually declined over time, potentially introducing selection bias. However, the NCHS has addressed this issue by employing enhanced weighting adjustments for NHANES data to minimize potential nonresponse bias. Finally, the NHANES lacks information on other risk factors for arthritis, limiting our ability to fully assess their impact on prevalence.

## Conclusions

This nationally representative survey provides robust data for understanding the significant trends in arthritis prevalence among U.S. adults. From 2011 to 2018, we observed variations in arthritis prevalence trends among different races. Across all indicators, rates were higher among White, Black, and other racial groups , with Black individuals showing a relatively stable prevalence without a statistically significant increase over the years. Additionally, we found that the prevalence of arthritis and RA increased only in the Asian population. This discovery contributes to a deeper understanding of the disparities in arthritis prevalence among different populations.

### Electronic supplementary material

Below is the link to the electronic supplementary material.

Supplementary Material 1

## Data Availability

Publicly available datasets were analyzed in this study. All data generated or analyzed during this study are included in this published article and the NHANES website (https://www.cdc.gov/nchs/nhanes/index.htm).
