# Supplementary material for: Trends in prevalence of arthritis by race among adults in the United States, 2011–2018
Source: BMC Public Health. 2024 Jun 5;24:1507. doi: 10.1186/s12889-024-18966-0 (PMC11151635; doi:10.1186/s12889-024-18966-0)
Supplement: Supplementary file 1 — Supplementary Material 1 [file 12889_2024_18966_MOESM1_ESM.docx]

**Supplemental Online Content**

**Supplementary Table S1** Population Characteristics for Participants: NHANES, 2011−18

**Supplementary Table S2** Unweighted Sample Sizes for Participants by Sex, Age Group, and Race: NHANES 2017−18

**Supplementary Table S3** Unweighted Sample Sizes for the Trend of Arthritis Prevalence, National Health and Nutrition Examination Survey, 2011−18 (*n* = 22,566)

**Supplementary Table S4** Unweighted Sample Sizes for the Trend of Osteoarthritis Prevalence, National Health and Nutrition Examination Survey, 2011−18 (*n* = 20,898)

**Supplementary Table S5** Unweighted Sample Sizes for the Trend of Rheumatoid Arthritis Prevalence, National Health and Nutrition Examination Survey, 2011−18 (*n* = 20,898)

**Supplementary Table S6** Unweighted Sample Sizes for the Trend of Psoriatic arthritis Prevalence, National Health and Nutrition Examination Survey, 2011−18 (*n* = 20,898)

**Supplementary Table S7** Unweighted Sample Sizes for the Trend of Other Arthritis Prevalence, National Health and Nutrition Examination Survey, 2011−18 (*n* = 20,898)

**Supplementary Figure S1** The prevalence and age trend of arthritis by sex (2011−18)

**Supplementary Table S1** Population Characteristics for Participants: NHANES, 2011−18

| **Variables** | **2011**−**12** | | **2013**−**14** | | **2015**−**16** | | **2017**−**18** | |
| --- | --- | --- | --- | --- | --- | --- | --- | --- |
|  | **Unweighted**  **No. of**  **participants** | **Weighted**  **percentage**  **(95% CI)** | **Unweighted**  **No. of**  **participants** | **Weighted**  **percentage**  **(95% CI)** | **Unweighted**  **No. of**  **participants** | **Weighted**  **percentage**  **(95% CI)** | **Unweighted**  **No. of**  **participants** | **Weighted**  **percentage**  **(95% CI)** |
| **Overall** | 5550 | / | 5756 | / | 5708 | / | 5552 | / |
| **Age, years** |  |  |  |  |  |  |  |  |
| 20−34 | 1395 | 25.92 (24.43, 27.48) | 1377 | 25.92 (24.56, 27.33) | 1373 | 26.29 (24.81, 27.83) | 1188 | 25.82 (24.15, 27.56) |
| 35−50 | 1458 | 29.11 (27.48, 30.79) | 1609 | 28.93 (27.50, 30.40) | 1516 | 27.38 (25.83, 28.99) | 1308 | 26.57 (24.81, 28.40) |
| 51−64 | 1357 | 25.61 (23.88, 27.42) | 1399 | 25.05 (23.57, 26.59) | 1365 | 24.76 (23.14, 26.46) | 1452 | 25.99 (24.13, 27.94) |
| 65−80 | 1340 | 19.36 (18.05, 20.74) | 1404 | 20.10 (18.90, 21.36) | 1454 | 21.56 (20.14, 23.05) | 1604 | 21.63 (20.16, 23.16) |
| **Sex** |  |  |  |  |  |  |  |  |
| Male | 2734 | 47.93 (46.10, 49.77) | 2752 | 48.07 (46.45, 49.69) | 2744 | 48.15 (46.36, 49.94) | 2697 | 48.15 (46.15, 50.17) |
| Female | 2816 | 52.07 (50.23, 53.90) | 3004 | 51.93 (50.31, 53.55) | 2964 | 51.85 (50.06, 53.64) | 2855 | 51.85 (49.83, 53.85) |
| **Race/ethnicity** |  |  |  |  |  |  |  |  |
| Mexican American | 539 | 7.66 (6.99, 8.39) | 765 | 9.17 (8.48, 9.91) | 993 | 8.84 (8.22, 9.50) | 733 | 8.82 (8.07, 9.62) |
| Other Hispanic | 577 | 6.61 (6.03, 7.25) | 507 | 5.54 (5.03, 6.10) | 767 | 6.45 (5.93, 7.00) | 513 | 6.92 (6.23, 7.67) |
| Non-Hispanic White | 2035 | 66.48 (65.02, 67.90) | 2468 | 65.78 (64.44, 67.10) | 1857 | 63.84 (62.36, 65.30) | 1931 | 62.37 (60.64, 64.06) |
| Non-Hispanic Black | 1455 | 11.82 (10.84, 12.24) | 1174 | 11.44 (10.75, 12.18) | 1197 | 11.36 (10.66, 12.10) | 1294 | 11.46 (10.70, 12.26) |
| Non-Hispanic Asian | 792 | 5.23 (4.84, 5.65) | 665 | 5.32 (4.90, 5.77) | 690 | 5.83 (5.38, 6.32) | 808 | 5.91 (4.45, 6.40) |
| Other Race | 152 | 2.50 (2.01, 3.12) | 177 | 2.74 (2.29, 3.27) | 204 | 3.67 (3.07, 4.38) | 273 | 4.54(3.76, 5.47) |

**Supplementary Table S2** Unweighted Sample Sizes for Participants by Sex, Age Group, and Race: NHANES 2017−18

| **Age groups, year** | **No. of Participants by Race/Ethnicity** | | | | | | |
| --- | --- | --- | --- | --- | --- | --- | --- |
|  | **Overall** | **Mexican American** | **Other Hispanic** | **Non-Hispanic White** | **Non-Hispanic Black** | **Non-Hispanic Asian** | **Other Race** |
| **All participants** |  |  |  |  |  |  |  |
| 20−34 | 1188 | 184 | 110 | 359 | 269 | 191 | 75 |
| 35−50 | 1308 | 203 | 112 | 369 | 302 | 225 | 70 |
| 51−64 | 1452 | 206 | 168 | 404 | 371 | 238 | 65 |
| 65−80 | 1604 | 140 | 123 | 772 | 352 | 154 | 63 |
| **Male participants** |  |  |  |  |  |  |  |
| 20−34 | 583 | 97 | 54 | 173 | 124 | 94 | 41 |
| 35−50 | 597 | 88 | 44 | 185 | 139 | 104 | 37 |
| 51−64 | 705 | 104 | 76 | 206 | 172 | 112 | 35 |
| 65−80 | 812 | 64 | 58 | 407 | 180 | 65 | 38 |
| **Female participants** |  |  |  |  |  |  |  |
| 20−34 | 605 | 87 | 56 | 186 | 145 | 97 | 34 |
| 35−50 | 711 | 115 | 68 | 211 | 163 | 121 | 33 |
| 51−64 | 747 | 102 | 92 | 198 | 199 | 126 | 30 |
| 65−80 | 792 | 76 | 65 | 365 | 172 | 89 | 25 |

**Supplementary Table S3** Unweighted Sample Sizes for the Trend of Arthritis Prevalence, National Health and Nutrition Examination Survey, 2011−18 (*n* = 22,566)

| **Variable** | **Prevalence, % (Unweighted)** | | | | |
| --- | --- | --- | --- | --- | --- |
|  | **2011**−**12** | **2013**−**14** | **2015**−**16** | **2017**−**18** | **P for trend** |
| **Overall** | 1365 (24.59%) | 1508 (26.20%) | 1470 (25.75%) | 1695 (30.53%) | **< 0.001** |
| **All participants** |  |  |  |  |  |
| Mexican American | 92 (17.07%) | 168 (21.96%) | 193 (19.44%) | 159 (21.69%) | 0.106 |
| Other Hispanic | 143 (24.78%) | 101 (19.92%) | 206 (26.86%) | 140 (27.29%) | **0.020** |
| Non-Hispanic White | 628 (30.86%) | 825 (33.43%) | 621 (33.44%) | 759 (39.31%) | **< 0.001** |
| Non-Hispanic Black | 395 (27.15%) | 313 (26.66%) | 306 (25.56%) | 409 (31.61%) | **0.004** |
| Non-Hispanic Asian | 78 (9.85%) | 63 (9.47%) | 78 (11.30%) | 128 (15.84%) | **< 0.001** |
| Other Race | 29 (19.08%) | 38 (21.47%) | 66 (32.35%) | 100 (36.63%) | **< 0.001** |
| **Age (yrs)** |  |  |  |  |  |
| 20−34 | 48 (3.44%) | 61 (4.43%) | 53 (3.86%) | 61 (5.13%) | 0.160 |
| 35−50 | 189 (12.96%) | 225 (13.98%) | 223 (14.71%) | 207 (15.83%) | 0.176 |
| 51−64 | 438 (32.28%) | 496 (36.31%) | 466 (34.14%) | 549 (37.81%) | **0.013** |
| 65−80 | 690 (51.49%) | 726 (51.71%) | 728 (50.07%) | 878 (54.74%) | 0.067 |
| Trend of affected age | 62.56 ± 13.83 | 61.35 ± 13.77 | 61.99 ± 13.79 | 62.91 ± 13.50 | **0.007** |
| **Male participants** |  |  |  |  |  |
| Mexican American | 37 (12.98%) | 66 (17.51%) | 79 (17.21%) | 60 (17.00%) | 0.380 |
| Other Hispanic | 46 (17.56%) | 33 (15.07%) | 67 (20.06%) | 48 (20.69%) | 0.371 |
| Non-Hispanic White | 270 (26.47%) | 318 (27.09%) | 274 (28.99%) | 348 (35.84%) | **< 0.001** |
| Non-Hispanic Black | 149 (21.35%) | 121 (20.90%) | 110 (19.57%) | 172 (27.97%) | **0.002** |
| Non-Hispanic Asian | 24 (6.20%) | 16 (5.02%) | 22 (6.51%) | 49 (13.07%) | **< 0.001** |
| Other Race | 13 (15.85%) | 14 (16.67%) | 35 (33.02%) | 48 (31.79%) | **0.004** |
| All male participants | 539 (19.71%) | 568 (20.64%) | 587 (21.39%) | 725 (26.88%) | **< 0.001** |
| Age (yrs) | 63.08 ± 13.63 | 61.04 ± 13.86 | 62.62 ± 13.52 | 62.98 ± 13.26 | 0.045 |
| **Female participants** |  |  |  |  |  |
| Mexican American | 55 (21.65%) | 102 (26.29%) | 114 (21.35%) | 99 (26.05%) | 0.187 |
| Other Hispanic | 97 (30.79%) | 68 (23.61%) | 139 (32.10%) | 92 (32.74%) | 0.055 |
| Non-Hispanic White | 358 (35.27%) | 507 (39.18%) | 347 (38.05%) | 411 (42.81%) | **0.007** |
| Non-Hispanic Black | 246 (32.50%) | 192 (32.27%) | 196 (30.87%) | 237 (34.90%) | 0.469 |
| Non-Hispanic Asian | 54 (13.33%) | 47 (13.58%) | 56 (15.91%) | 79 (18.24%) | 0.174 |
| Other Race | 16 (22.86%) | 24 (25.81%) | 31 (31.63%) | 52 (42.62%) | **0.014** |
| All female participants | 826 (29.33%) | 940 (31.29%) | 883 (29.79%) | 970 (33.98%) | **< 0.001** |
| Age (yrs) | 62.22 ± 13.96 | 61.53 ± 13.72 | 61.57 ± 13.96 | 62.86 ± 13.68 | 0.096 |

**Supplementary Table S4** Unweighted Sample Sizes for the Trend of Osteoarthritis Prevalence, National Health and Nutrition Examination Survey, 2011−18 (*n* = 20,898)

| **Variable** | **Prevalence, % (Unweighted)** | | | | |
| --- | --- | --- | --- | --- | --- |
|  | **2011-2012** | **2013-2014** | **2015-2016** | **2017-2018** | **P for trend** |
| **Overall** | 576 (11.23%) | 694 (12.96%) | 616 (11.61%) | 711 (13.92%) | **< 0.001** |
| **All participants** |  |  |  |  |  |
| Mexican American | 31 (6.13%) | 58 (8.12%) | 66 (7.09%) | 49 (7.27%) | 0.615 |
| Other Hispanic | 52 (9.89%) | 36 (7.68%) | 61 (8.57%) | 44 (9.50%) | 0.614 |
| Non-Hispanic White | 337 (17.89%) | 457 (19.88%) | 308 (18.03%) | 391 (22.10%) | **0.004** |
| Non-Hispanic Black | 106 (8.16%) | 104 (9.82%) | 106 (9.67%) | 129 (10.98%) | 0.125 |
| Non-Hispanic Asian | 33 (4.29%) | 24 (3.73%) | 41 (6.15%) | 47 (6.09%) | 0.084 |
| Other Race | 17 (11.64%) | 15 (8.82%) | 34 (17.71%) | 51 (20.16%) | **0.006** |
| **Age (yrs)** |  |  |  |  |  |
| 20−34 | 20 (1.45%) | 27 (1.98%) | 13 (0.96%) | 18 (1.54%) | 0.181 |
| 35−50 | 71 (5.04%) | 89 (5.73%) | 81 (5.54%) | 78 (6.22%) | 0.617 |
| 51−64 | 170 (13.95%) | 219 (17.62%) | 189 (15.28%) | 224 (17.09%) | **0.049** |
| 65−80 | 315 (28.07%) | 359 (30.04%) | 333 (26.60%) | 391 (28.52%) | 0.304 |
| Trend of affected age | 63.59 ± 13.86 | 62.51 ± 13.46 | 63.30 ± 13.16 | 64.20 ± 13.21 | 0.104 |
| **Male participants** |  |  |  |  |  |
| Mexican American | 11 (4.07%) | 25 (6.93%) | 21 (4.87%) | 12 (3.66%) | 0.204 |
| Other Hispanic | 20 (8.03%) | 9 (4.35%) | 23 (7.21%) | 10 (4.72%) | 0.266 |
| Non-Hispanic White | 127 (13.51%) | 159 (14.51%) | 113 (13.12%) | 150 (17.06%) | 0.082 |
| Non-Hispanic Black | 36 (5.68%) | 37 (6.92%) | 30 (5.76%) | 51 (8.99%) | 0.092 |
| Non-Hispanic Asian | 10 (2.62%) | 10 (3.16%) | 13 (3.88%) | 11 (3.06%) | 0.814 |
| Other Race | 5 (6.49%) | 7 (8.43%) | 16 (16.49%) | 26 (18.44%) | **0.033** |
| All male participants | 209 (8.19%) | 247 (9.51%) | 216 (8.42%) | 260 (10.45%) | **0.019** |
| Age (yrs) | 62.63 ± 14.42 | 61.77 ± 13.58 | 62.93 ± 13.57 | 63.40 ± 13.42 | 0.531 |
| **Female participants** |  |  |  |  |  |
| Mexican American | 20 (8.47%) | 33 (9.35%) | 45 (9.00%) | 37 (10.69%) | 0.798 |
| Other Hispanic | 32 (11.55%) | 27 (10.31%) | 38 (9.67%) | 34 (13.55%) | 0.463 |
| Non-Hispanic White | 210 (22.25%) | 298 (24.77%) | 195 (23.02%) | 241 (27.08%) | 0.079 |
| Non-Hispanic Black | 70 (10.53%) | 67 (12.79%) | 76 (13.22%) | 78 (12.83%) | 0.446 |
| Non-Hispanic Asian | 23 (5.93%) | 14 (4.27%) | 28 (8.43%) | 36 (8.74%) | 0.058 |
| Other Race | 12 (17.39%) | 8 (9.20%) | 18 (18.95%) | 25 (22.32%) | 0.105 |
| All female participants | 367 (14.23%) | 447 (16.21%) | 400 (14.59%) | 451 (17.22%) | **0.008** |
| Age (yrs) | 64.14 ± 13.51 | 62.93 ± 13.40 | 63.49 ± 12.94 | 64.65 ± 13.09 | 0.214 |

**Supplementary Table S5** Unweighted Sample Sizes for the Trend of Rheumatoid Arthritis Prevalence, National Health and Nutrition Examination Survey, 2011−18 (*n* = 20,898)

| **Variable** | **Prevalence, % (Unweighted)** | | | | |
| --- | --- | --- | --- | --- | --- |
|  | **2011**−**12** | **2013**−**14** | **2015**−**16** | **2017**−**18** | **P for trend** |
| **Overall** | 249 (4.85%) | 254 (4.74%) | 292 (5.50%) | 337 (6.60%) | **< 0.001** |
| **All participants** |  |  |  |  |  |
| Mexican American | 22 (4.35%) | 43 (6.02%) | 51 (5.48%) | 39 (5.79%) | 0.620 |
| Other Hispanic | 25 (4.75%) | 12 (2.56%) | 59 (8.29%) | 30 (6.48%) | **< 0.001** |
| Non-Hispanic White | 82 (4.35%) | 122 (5.31%) | 88 (5.15%) | 105 (5.94%) | 0.190 |
| Non-Hispanic Black | 97 (7.47%) | 62 (5.85%) | 74 (6.75%) | 113 (9.62%) | **0.005** |
| Non-Hispanic Asian | 18 (2.34%) | 9 (1.40%) | 6 (0.90%) | 30 (3.89%) | **< 0.001** |
| Other Race | 5 (3.42%) | 6 (3.53%) | 14 (7.29%) | 20 (7.91%) | 0.121 |
| **Age (yrs)** |  |  |  |  |  |
| 20−34 | 4 (0.29%) | 12 (0.88%) | 13 (0.96%) | 13 (1.11%) | 0.088 |
| 35−50 | 44 (3.12%) | 41 (2.64%) | 45 (3.08%) | 39 (3.11%) | 0.840 |
| 51−64 | 89 (7.30%) | 94 (7.56%) | 101 (8.16%) | 117 (8.92%) | 0.440 |
| 65−80 | 112 (9.98%) | 107 (8.95%) | 133 (10.62%) | 168 (12.25%) | **0.049** |
| Trend of affected age | 61.10 ± 13.08 | 59.63 ± 13.95 | 61.03 ± 13.65 | 62.50 ± 12.92 | 0.079 |
| **Male participants** |  |  |  |  |  |
| Mexican American | 9 (3.33%) | 17 (4.71%) | 22 (5.10%) | 15 (4.57%) | 0.741 |
| Other Hispanic | 6 (2.41%) | 4 (1.93%) | 18 (5.64%) | 12 (5.66%) | 0.055 |
| Non-Hispanic White | 37 (3.94%) | 44 (4.01%) | 42 (4.88%) | 57 (6.48%) | **0.036** |
| Non-Hispanic Black | 37 (5.84%) | 28 (5.23%) | 28 (5.37%) | 50 (8.82%) | **0.045** |
| Non-Hispanic Asian | 6 (1.57%) | 1 (0.32%) | 4 (1.19%) | 14 (3.89%) | **0.003** |
| Other Race | 2 (2.60%) | 0 (0.00%) | 6 (6.19%) | 9 (6.38%) | 0.084 |
| All male participants | 97 (3.80%) | 94 (3.62%) | 120 (4.68%) | 157 (6.31%) | **< 0.001** |
| Age (yrs) | 62.04 ± 13.06 | 59.03 ± 13.46 | 62.51 ± 12.63 | 63.20 ± 13.17 | 0.082 |
| **Female participants** |  |  |  |  |  |
| Mexican American | 13 (5.51%) | 26 (7.37%) | 29 (5.80%) | 24 (6.94%) | 0.723 |
| Other Hispanic | 19 (6.86%) | 8 (3.05%) | 41 (10.43%) | 18 (7.17%) | **0.005** |
| Non-Hispanic White | 45 (4.77%) | 78 (6.48%) | 46 (5.43%) | 48 (5.39%) | 0.371 |
| Non-Hispanic Black | 60 (9.02%) | 34 (6.49%) | 46 (8.00%) | 63 (10.36%) | 0.121 |
| Non-Hispanic Asian | 12 (3.09%) | 8 (2.44%) | 2 (0.60%) | 16 (3.88%) | **0.040** |
| Other Race | 3 (4.35%) | 6 (6.90%) | 8 (8.42%) | 11 (9.82%) | 0.584 |
| All female participants | 152 (5.89%) | 160 (5.80%) | 172 (6.27%) | 180 (6.87%) | 0.359 |
| Age (yrs) | 60.49 ± 13.10 | 59.98 ± 14.26 | 60.01 ± 14.26 | 61.89 ± 12.70 | 0.573 |

**Supplementary Table S6** Unweighted Sample Sizes for the Trend of Psoriatic arthritis Prevalence, National Health and Nutrition Examination Survey, 2011−18 (*n* = 20,898)

| **Variable** | **Prevalence, % (Unweighted)** | | | | |
| --- | --- | --- | --- | --- | --- |
|  | **2011**−**12** | **2013**−**14** | **2015**−**16** | **2017**−**18** | **P for trend** |
| **Overall** | 17 (0.33%) | 21 (0.39%) | 18 (0.34%) | 26 (0.51%) | 0.448 |
| **All participants** |  |  |  |  |  |
| Mexican American | 1 (0.20%) | 3 (0.42%) | 2 (0.21%) | 3 (0.45%) | 0.772 |
| Other Hispanic | 2 (0.38%) | 4 (0.85%) | 2 (0.28%) | 2 (0.43%) | 0.540 |
| Non-Hispanic White | 7 (0.37%) | 11 (0.48%) | 12 (0.70%) | 11 (0.62%) | 0.525 |
| Non-Hispanic Black | 7 (0.54%) | 1 (0.09%) | 2 (0.18%) | 7 (0.60%) | 0.117 |
| Non-Hispanic Asian | - | 2 (0.31%) | - | 2 (0.26%) | 0.248 |
| Other Race | - | - | - | 1 (0.40%) | 0.570 |
| **Age (yrs)** |  |  |  |  |  |
| 20−34 | 1 (0.07%) | 1 (0.07%) | - | 1 (0.09%) | 0.902 |
| 35−50 | 6 (0.43%) | 5 (0.32%) | 7 (0.48%) | 6 (0.48%) | 0.157 |
| 51−64 | 4 (0.33%) | 9 (0.72%) | 3 (0.24%) | 10 (0.76%) | 0.946 |
| 65−80 | 17 (0.33%) | 21 (0.39%) | 18 (0.34%) | 26 (0.51%) | 0.448 |
| Trend of affected age | 56.29 ± 14.20 | 56.24 ± 14.99 | 58.39 ± 14.46 | 56.96 ± 11.75 | 0.983 |
| **Male participants** |  |  |  |  |  |
| Mexican American | 1 (0.37%) | 1 (0.28%) | 2 (0.46%) | 3 (0.91%) | 0.663 |
| Other Hispanic | 1 (0.40%) | 1 (0.48%) | 2 (0.63%) | - | 0.733 |
| Non-Hispanic White | 3 (0.32%) | 6 (0.55%) | 7 (0.81%) | 7 (0.80%) | 0.480 |
| Non-Hispanic Black | 2 (0.32%) | - | - | 3 (0.53%) | 0.170 |
| Non-Hispanic Asian | - | - | - | 1 (0.28%) | 0.412 |
| Other Race | - | - | - | - | - |
| All male participants | 7 (0.27%) | 8 (0.31%) | 11 (0.43%) | 14 (0.56%) | 0.344 |
| Age (yrs) | 56.00 ± 12.86 | 51.25 ± 15.17 | 60.36 ± 15.98 | 59.21 ± 8.97 | 0.409 |
| **Female participants** |  |  |  |  |  |
| Mexican American | - | 2 (0.57%) | - | - | 0.105 |
| Other Hispanic | 1 (0.36%) | 3 (1.15%) | - | 2 (0.80%) | 0.199 |
| Non-Hispanic White | 4 (0.42%) | 5 (0.42%) | 5 (0.59%) | 4 (0.45%) | 0.942 |
| Non-Hispanic Black | 5 (0.75%) | 1 (0.19%) | 2 (0.35%) | 4 (0.66%) | 0.494 |
| Non-Hispanic Asian | - | 2 (0.61%) | - | 1 (0.24%) | 0.248 |
| Other Race | - | - | - | 1 (0.89%) | 0.523 |
| All female participants | 10 (0.39%) | 13 (0.47%) | 7 (0.26%) | 12 (0.46%) | 0.562 |
| Age (yrs) | 56.50 ± 15.76 | 59.31 ± 14.61 | 55.29 ± 12.18 | 54.33 ± 14.30 | 0.810 |

**Supplementary Table S7** Unweighted Sample Sizes for the Trend of Other Arthritis Prevalence, National Health and Nutrition Examination Survey, 2011−18 (*n* = 20,898)

| **Variable** | **Prevalence, % (Unweighted)** | | | | |
| --- | --- | --- | --- | --- | --- |
|  | **2011**−**12** | **2013**−**14** | **2015**−**16** | **2017**−**18** | **P for trend** |
| **Overall** | 104 (2.03%) | 138 (2.58%) | 142 (2.68%) | 175 (3.43%) | **<0.001** |
| **All participants** |  |  |  |  |  |
| Mexican American | 5 (0.99%) | 13 (1.82%) | 12 (1.29%) | 9 (1.34%) | 0.647 |
| Other Hispanic | 13 (2.47%) | 11 (2.35%) | 29 (4.07%) | 14 (3.02%) | 0.278 |
| Non-Hispanic White | 51 (2.71%) | 66 (2.87%) | 64 (3.75%) | 90 (5.09%) | **< 0.001** |
| Non-Hispanic Black | 29 (2.23%) | 31 (2.93%) | 23 (2.10%) | 41 (3.49%) | 0.132 |
| Non-Hispanic Asian | 5 (0.65%) | 7 (1.09%) | 8 (1.20%) | 13 (1.68%) | 0.302 |
| Other Race | 1 (0.68%) | 10 (5.88%) | 6 (3.12%) | 8 (3.16%) | 0.081 |
| **Age (yrs)** |  |  |  |  |  |
| 20−34 | 9 (0.65%) | 8 (0.59%) | 8 (0.59%) | 10 (0.86%) | 0.829 |
| 35−50 | 19 (1.35%) | 34 (2.19%) | 37 (2.53%) | 31 (2.47%) | 0.113 |
| 51−64 | 37 (3.04%) | 51 (4.10%) | 45 (3.64%) | 57 (4.35%) | 0.330 |
| 65−80 | 39 (3.48%) | 45 (3.77%) | 52 (4.15%) | 77 (5.62%) | **0.037** |
| Trend of affected age | 57.97 ± 15.40 | 56.31 ± 14.19 | 57.98 ± 14.71 | 60.41 ± 14.78 | 0.080 |
| **Male participants** |  |  |  |  |  |
| Mexican American | 1 (0.37%) | 7 (1.94%) | 6 (1.39%) | 5 (1.52%) | 0.403 |
| Other Hispanic | 6 (2.41%) | 7 (3.38%) | 9 (2.82%) | 6 (2.83%) | 0.943 |
| Non-Hispanic White | 23 (2.45%) | 31 (2.83%) | 28 (3.25%) | 42 (4.78%) | **0.030** |
| Non-Hispanic Black | 10 (1.58%) | 12 (2.24%) | 11 (2.11%) | 20 (3.53%) | 0.155 |
| Non-Hispanic Asian | 3 (0.79%) | 2 (0.63%) | 2 (0.60%) | 8 (2.22%) | 0.109 |
| Other Race | 1 (1.30%) | 6 (7.23%) | 4 (4.12%) | 3 (2.13%) | 0.145 |
| All male participants | 44 (1.72%) | 65 (2.50%) | 60 (2.34%) | 84 (3.38%) | **0.002** |
| Age (yrs) | 60.39 ± 14.10 | 57.31 ± 14.84 | 57.08 ± 14.12 | 61.96 ± 12.96 | 0.158 |
| **Female participants** |  |  |  |  |  |
| Mexican American | 4 (1.69%) | 6 (1.70%) | 6 (1.20%) | 4 (1.16%) | 0.879 |
| Other Hispanic | 7 (2.53%) | 4 (1.53%) | 20 (5.09%) | 8 (3.19%) | 0.070 |
| Non-Hispanic White | 28 (2.97%) | 35 (2.91%) | 36 (4.25%) | 48 (5.39%) | **0.011** |
| Non-Hispanic Black | 19 (2.86%) | 19 (3.63%) | 12 (2.09%) | 21 (3.45%) | 0.418 |
| Non-Hispanic Asian | 2 (0.52%) | 5 (1.52%) | 6 (1.81%) | 5 (1.21%) | 0.429 |
| Other Race | - | 4 (4.60%) | 2 (2.11%) | 5 (4.46%) | 0.268 |
| All female participants | 60 (2.33%) | 73 (2.65%) | 82 (2.99%) | 91 (3.47%) | 0.078 |
| Age (yrs) | 56.20 ± 16.17 | 55.42 ± 13.62 | 58.63 ± 15.18 | 58.97 ± 16.23 | 0.328 |


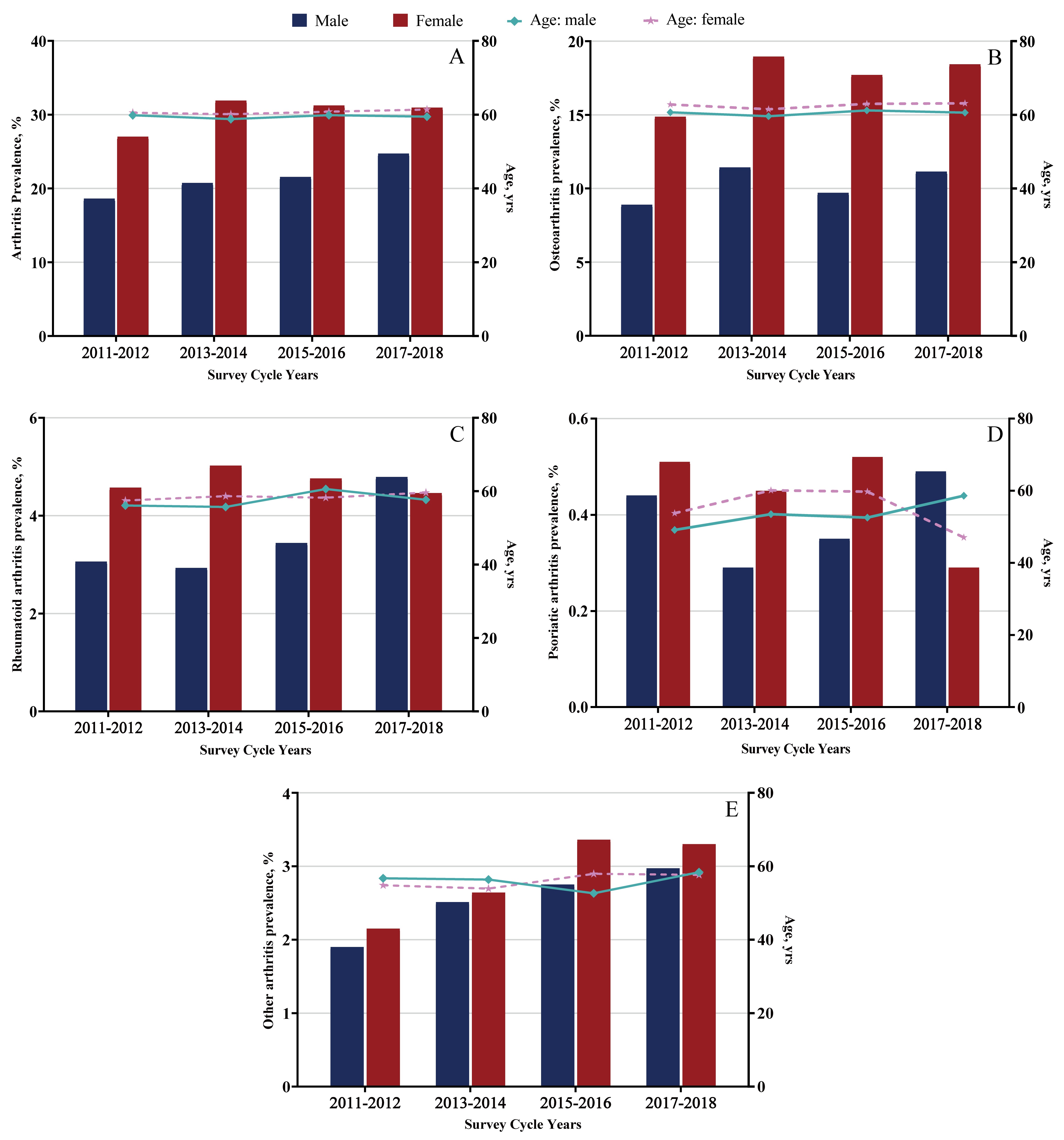


**Supplementary Figure S1** The prevalence and age trend of arthritis by sex (2011−18)
